# Mesenteric Panniculitis Presenting With Atypical Abdominal Pain: A Case Report

**DOI:** 10.7759/cureus.91402

**Published:** 2025-09-01

**Authors:** Andreas Koumenis, Alexandros Manthas, Theodoros Piperos, Theodoros Mariolis-Sapsakos

**Affiliations:** 1 Laboratory of Anatomy, Faculty of Nursing, National and Kapodistrian University of Athens, Athens, GRC; 2 Evgenidio Hospital, National and Kapodistrian University of Athens, Athens, GRC

**Keywords:** abdominal pain, computed tomography, glucocorticoids, mesenteric panniculitis, misty mesentery, sclerosing mesenteritis, weight loss

## Abstract

Mesenteric panniculitis (MP) is a rare, idiopathic inflammatory disorder of the mesenteric adipose tissue that often mimics other GI conditions, including malignancy, making diagnosis challenging. We present the case of a 77-year-old woman with a history of atrial fibrillation, hypertension, and mild heart failure who was admitted with an eight-day history of abdominal pain localized to the hypogastric region and unintentional weight loss of 6 kg over four months. On admission, her vital signs were stable, and laboratory tests, including CRP, were within normal limits. Abdominal X-ray revealed no abnormalities; however, CT demonstrated a “misty mesentery” appearance with scattered lymphadenopathy, consistent with MP. Colonoscopy excluded malignancy and other colonic pathology, and biopsy was deemed unnecessary. The patient was treated with oral glucocorticoids and analgesics, which led to substantial relief of abdominal symptoms within 72 hours. At three-month follow-up, she reported resolution of abdominal pain, weight gain, and stable laboratory results. A six-month CT scan demonstrated stable mesenteric findings, and she remains under annual surveillance. This case highlights the diagnostic challenge of MP due to its nonspecific clinical manifestations and frequent overlap with malignant conditions. CT is the imaging modality of choice, with characteristic features such as “misty mesentery” or “fat-ring sign” aiding differentiation. Most cases are managed conservatively, with glucocorticoids providing effective symptom relief. Awareness of this entity among clinicians is essential to prevent misdiagnosis and avoid unnecessary invasive investigations or surgical interventions.

## Introduction

Mesenteric panniculitis (MP) is a rare, benign, chronic inflammatory, and fibrosing disorder affecting the mesenteric adipose tissue [1]. Although any GI mesentery may be involved, the small bowel mesentery is most frequently affected [1]. Histologically, MP represents a spectrum of chronic inflammation, fat necrosis, and fibrosis [2], described in three stages: mesenteric lipodystrophy, MP, and retractile mesenteritis [1]. Clinical manifestations depend on disease stage, ranging from asymptomatic incidental findings to severe complications such as bowel obstruction. Despite its “benign” classification, MP can cause significant morbidity, with symptoms including abdominal pain, fever, weight loss, or complications such as chylous effusions [1].

For clinicians, the challenge lies in distinguishing MP from malignant conditions such as lymphoma or peritoneal carcinomatosis. Epidemiologically, MP is rare, with reported incidence ranging from 0.6% to 3.4% in the literature [3-7]. It usually presents between the fifth and seventh decades, with a mean age of onset around 60-65 years, and occurs more often in men, with male-to-female ratios of 2:1 to 3:1 [1]. The current case of a 77-year-old woman deviates from typical demographic expectations, highlighting the importance of recognizing MP outside its usual profile.

The etiology remains uncertain. Suggested triggers include prior surgery, abdominal trauma, autoimmune disease, infection, and paraneoplastic associations [1]. Pathogenesis likely involves inflammation, fat necrosis, and fibrosis [2]. Because no specific markers exist, MP is largely a diagnosis of exclusion, with malignancy serving both as a differential diagnosis and a potential associated factor.

Clinical presentation varies. Between 30% and 50% of patients are asymptomatic, while abdominal pain occurs in 30-50% [3]. Other features include palpable mass, nausea, vomiting, fever, weight loss, constipation, or diarrhea [3]. Rare complications include ascites or bowel obstruction [1]. CT is the diagnostic modality of choice [1], with findings such as increased mesenteric fat attenuation and characteristic signs like the “fat ring sign” or “tumor pseudocapsule” [5]. Because radiological overlap with malignancy is common, histopathology is often required for confirmation [1].

This case is clinically significant because it combines three uncommon features: the rarity of MP itself, its presentation as atypical abdominal pain, and its occurrence in a 77-year-old woman, diverging from the typical male predominance and mean age of 60-65 years.

## Case presentation

A 77-year-old woman was admitted to our ED and referred to the surgical unit with abdominal pain primarily located in the hypogastric region, which had started eight days prior. Her surgical history included cholecystectomy, and her medical history included atrial fibrillation, hypertension, and mild heart failure, for which she was taking furosemide, an anticoagulant, and a β-blocker. Three days earlier, she had visited the ED, reporting the same vague sub-umbilical pain, and was evaluated by the on-call internal medicine physician. At that time, no definitive diagnosis could be made, and admission was suggested, which she declined. She was treated with antibiotics for a urinary tract infection, based on a positive urinalysis and dysuria, and discharged with instructions to return if symptoms persisted or worsened. She also reported an unintentional weight loss of approximately 6 kg over four months.

**Figure 1:**
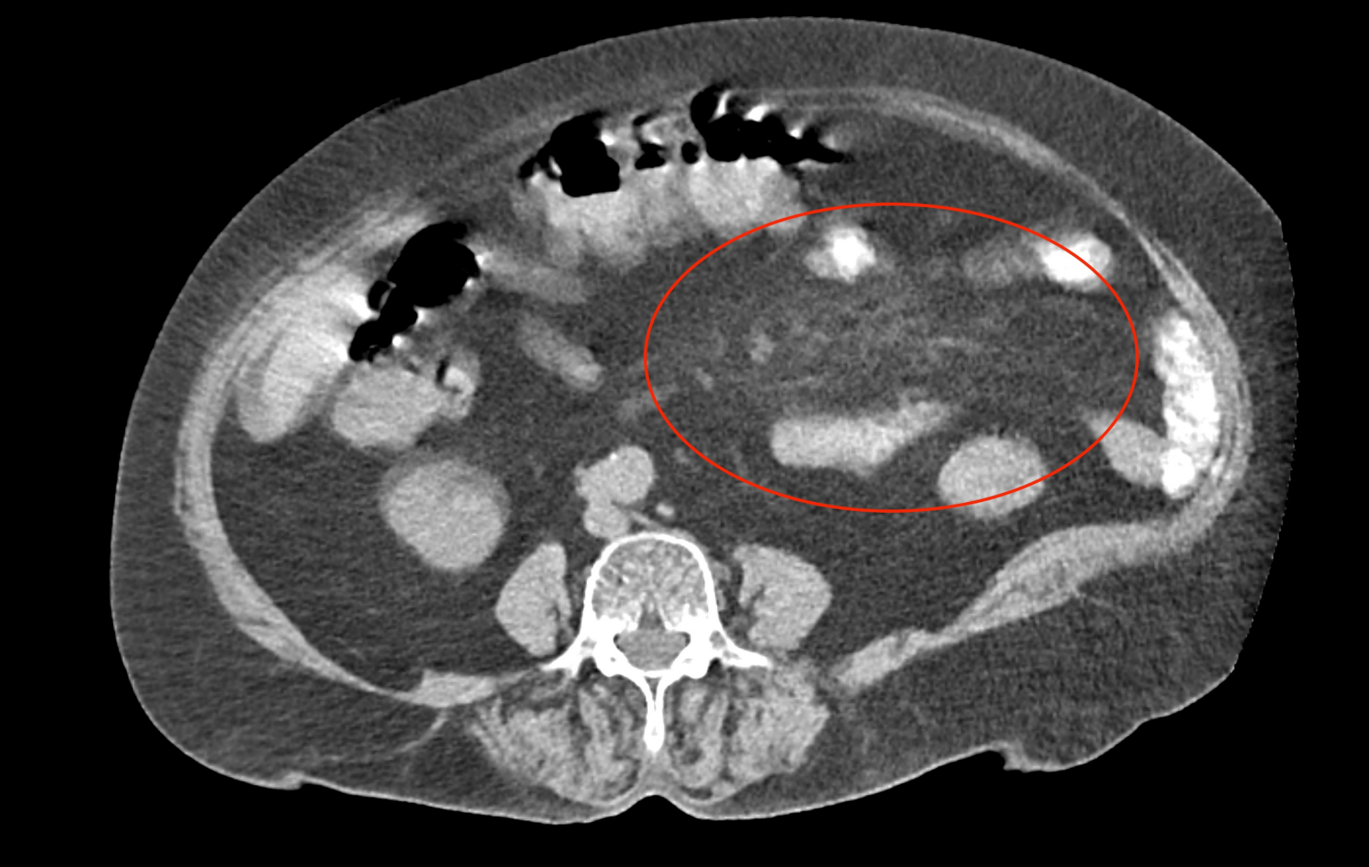
CT of the abdomen demonstrating the “misty mesentery” appearance Transverse view showing increased attenuation of mesenteric fat with ill-defined borders.

On admission, her vital signs were stable, and she was afebrile. Clinical examination revealed diffuse abdominal tenderness with mild distention. Digital rectal examination was unremarkable. Laboratory tests were all within normal limits, including CRP (0.25 mg/dL; normal range: <0.5 mg/dL); erythrocyte sedimentation rate (ESR) was not routinely assessed in our ED. Abdominal X-ray showed no significant findings. Given the chronicity of her pain, previous hospital visit, and current clinical findings, a CT scan was performed. Imaging revealed fatty haziness, consistent with a “misty mesentery” appearance, without any additional pathological findings (Figure 1).

**Figure 2:**
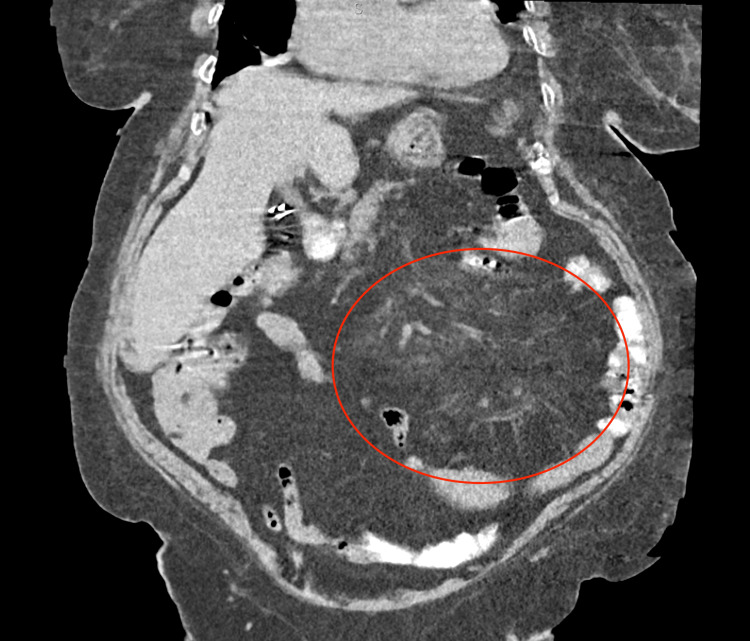
CT of the abdomen Coronal view showing diffuse mesenteric haziness with scattered lymph nodes, consistent with MP. MP, mesenteric panniculitis

Scattered enlarged lymph nodes were also present on imaging (Figure 2).

As these findings were highly suggestive of MP, a biopsy was not performed, and the diagnosis was established. A colonoscopy was conducted to rule out inflammation or malignancy, yielding normal results. The patient was started on oral glucocorticoids and analgesics and referred to the hospital’s gastroenterology clinic for follow-up and corticosteroid tapering. Within 72 hours of initiating glucocorticoid therapy, her abdominal symptoms improved substantially. At three-month follow-up, she reported complete resolution of abdominal pain, gradual weight gain, and normal laboratory results. A CT scan at six-month follow-up demonstrated stable mesenteric changes. The patient is scheduled to continue annual monitoring.

## Discussion

MP is a rare inflammatory disorder first described by Jura et al. in 1924 as “retractile mesenteritis” [1]. It primarily affects the mesentery, the connective tissue that supports and suspends the small intestine to the posterior abdominal wall, although cases involving the mesocolon have also been reported [1]. MP is characterized by inflammation, degeneration, and scarring of mesenteric adipose tissue. One study described MP as “a stage in the evolution of idiopathic disorders of the mesentery and peritoneum called sclerosing mesenteritis,” based on histologic findings, with mesenteric lipodystrophy as the initial stage, during which macrophages replace mesenteric fat [2]. The second stage, MP, involves infiltration of the adipose tissue by plasma cells, polymorphonuclear leukocytes, and lipid-laden macrophages. The final stage, retractile mesenteritis, is marked by varying degrees of fibrosis, likely secondary to necrotic fat, scattered lymphocytic aggregates, and lymphoid follicles, leading to tissue retraction [2]. Overall, terminology in the literature tends to overlap in the absence of biopsy confirmation.

The incidence of MP is low and variable in the literature. Differences between studies may result from misreporting by radiologists or selection bias at certain centers. Several studies analyzing large CT scan databases report very low prevalence rates [4-6], likely due to underreporting or mislabeling of findings, or failure to recognize features of MP. A study by Daskalogiannaki et al. evaluated over 7,500 CT scans for MP and reported an incidence of 0.6% [7]. More recent studies by Coulier and van Putte-Katier et al. reported higher incidences of 3.42% and 2.5%, respectively [8,9], which is also supported by Sharma et al.’s 2017 systematic review [10]. The higher rates may reflect advances in CT imaging technology, suggesting that MP, while uncommon, may be more prevalent than previously thought. Studies consistently report a male predominance, with male-to-female ratios of 2:1 to 3:1, typically in the fifth or sixth decade of life [1]. Several case reports, including ours, describe MP in elderly patients, emphasizing the need for clinical suspicion even outside typical demographics.

The etiology of MP remains uncertain, though several triggers have been suggested, including bacterial infections, autoimmune disorders, mesenteric ischemia, trauma, prior abdominal surgery, vasculitis, malignancy, and other granulomatous diseases. While earlier studies suggested an association between MP and neoplastic diseases, raising the possibility of a paraneoplastic phenomenon, a large case-control study of over 13,000 CT scans found no significant association between MP and malignancy [11]. Nevertheless, the literature remains divided on this issue.

Clinical presentation varies. Common symptoms include abdominal pain, bloating, diarrhea, nausea, vomiting, fever, and weight loss [3,12]. Abdominal pain is most frequent, typically described as dull or aching, intermittent or constant, of varying intensity, located in the upper or lower abdomen, and sometimes associated with tenderness on palpation. In some patients, pain intensity may correlate with bowel movements. MP can present acutely, with a sudden onset of abdominal pain, or chronically, with gradual symptom onset often associated with fibrosis and scarring, potentially leading to obstruction.

Diagnosis is challenging due to a nonspecific clinical presentation and the absence of a specific diagnostic test. Laboratory findings are usually nonspecific, though some patients may exhibit elevated inflammatory markers such as CRP or ESR, which are not unique to MP. Therefore, imaging, most commonly CT, is essential for diagnosis. Characteristic findings include a soft-tissue mass with a “misty mesentery” appearance due to mesenteric fat inflammation and fibrosis. The mass may envelop mesenteric vessels (the “fat ring sign”), aiding differentiation from lymphoma, carcinoid tumors, or metastases. Central necrotic areas with calcifications may also be present. Biopsy is sometimes necessary to confirm the diagnosis and exclude other conditions [13,14].

Management depends on symptom severity and disease extent. Asymptomatic patients may require only observation, while symptomatic patients are treated to relieve symptoms and prevent complications. Mild cases can be managed with nonsteroidal anti-inflammatory drugs and corticosteroids. More severe or refractory cases may require immunosuppressive therapy [12,15].

Although MP is generally benign with a favorable long-term prognosis, a 2016 study by Scheer et al. reported a five-fold higher risk of malignancy, predominantly non-Hodgkin lymphoma, in patients with MP [16]. Surgical intervention is reserved for advanced complications, such as fibrosis or intestinal obstruction, which may necessitate partial or complete resection of the affected mesentery or removal of associated masses. Cortés et al. reported that, among 103 biopsy-proven MP patients, only 5 (4.9%) required surgery for bowel resection or adhesiolysis [17]. Of these patients, 41.7% received medical therapy, most commonly prednisone plus tamoxifen [17]. In another series by Akram et al., 44 of 92 patients received treatment, and 20 underwent surgery, of whom 12 had surgery without subsequent medical therapy [12].

## Conclusions

MP is a rare and challenging diagnosis due to its nonspecific clinical manifestations and imaging findings. Although generally a benign condition, it can mimic malignancy, making careful evaluation and differentiation from other diseases essential. While most cases are idiopathic, some studies suggest associations with other medical conditions. CT is the preferred imaging modality, and characteristic findings, such as the “fat-ring” sign, can help distinguish MP from other disorders. Biopsy is not routinely required but may be performed to establish a definitive diagnosis. Management is usually conservative, with surgery reserved for complications or cases refractory to medical therapy.

Diagnosis can be difficult and typically requires a combination of laboratory and imaging studies. Clinical manifestations are often nonspecific; therefore, surgeons in the emergency setting should be aware of MP’s presentation to avoid misdiagnosis. MP may present acutely with sharp, nonspecific abdominal pain, particularly in older patients. This case emphasizes the importance of considering MP in elderly patients with unexplained abdominal pain and weight loss, as timely recognition can prevent unnecessary invasive procedures and ensure appropriate follow-up.
